# Chronic high-fat diet feeding reduces the rate of postprandial triglyceride absorption independent of intestinal ANGPTL4

**DOI:** 10.1016/j.jlr.2026.101124

**Published:** 2026-08-14

**Authors:** Shwetha K. Shetty, Kelli L. Sylvers-Davie, Brandon S.J. Davies

**Affiliations:** Department of Biochemistry and Molecular Biology, Fraternal Order of Eagles Diabetes Research Center, and Obesity Research and Education Initiative, University of Iowa, Iowa City, IA, USA

**Keywords:** pancreatic lipase, fat absorption, triglycerides, high-fat feeding

## Abstract

Pancreatic lipase is the major enzyme responsible for breaking down dietary triglycerides in the intestines. A previous report suggested that intestinal angiopoietin-like 4 (ANGPTL4) might serve as an endogenous inhibitor of pancreatic lipase and thus regulate fat absorption. As *Angptl4* expression is reportedly induced by high-fat-diet feeding, we hypothesized that induction of ANGPTL4 by a high-fat diet would lead to an increased inhibition of pancreatic lipase, less breakdown of dietary triglycerides, and ultimately a reduced rate of postprandial triglyceride absorption. To test this hypothesis, we generated intestinal epithelial cell–specific ANGPTL4 knockout mice, fed them diets with varying levels of fat, and measured postprandial triglyceride absorption and intestinal triglyceride lipase activity. As we hypothesized, we found that chronic high-fat feeding reduced the rate of postprandial triglyceride absorption in mice. However, this regulation of postprandial triglyceride absorption appeared to be largely independent of ANGPTL4, as similar decreases were observed in both wild-type and intestinal epithelial cell–specific ANGPTL4 knockout mice. We conclude that there is a mechanism by which chronic high-fat feeding reduces the rate of secretion of dietary triglycerides into the circulation, but that this mechanism does not require intestinal ANGPTL4.

Elevated plasma triglycerides (hypertriglyceridemia) can cause shifts in fatty acid delivery that lead to skeletal muscle insulin resistance, cardiac lipotoxicity, and fatty liver and often accompany metabolic diseases such as obesity, metabolic syndrome, and type 2 diabetes mellitus. Levels of plasma triglycerides are controlled by the balance of triglyceride entry into the circulation (either through dietary fat absorption in the gut or triglyceride synthesis and secretion from the liver) and triglyceride clearance (through the hydrolysis of plasma triglycerides by lipoprotein lipase or the uptake of lipoprotein remnant particles in the liver).

Dietary fat absorption begins with the hydrolysis of dietary triglycerides in the gut. While lingual lipase, gastric lipase, and bile-acid–induced lipase all contribute to fat digestion, pancreatic lipase acting in the small intestine is responsible for the majority of dietary triglyceride hydrolysis (1, 2, 3). The fatty acids released by hydrolysis are taken up by enterocytes where they are re-esterified and packaged into chylomicrons. These chylomicrons are then released into the circulation via the lymphatic system.

The role of angiopoietin-like 4 (ANGPTL4) in triglyceride clearance has been studied extensively, but its role in fat absorption remains unclear. ANGPTL4 regulates triglyceride clearance through inhibition of lipoprotein lipase. Systemic loss of ANGPTL4 function reduces plasma triglyceride levels in both mice and humans (4, 5, 6, 7). ANGPTL4 is expressed in multiple tissues including white and brown adipose tissue, liver, heart, and skeletal muscle (8, 9, 10). Tissue-specific inhibition of lipoprotein lipase by ANGPTL4 reduces adipose storage of triglycerides during fasting (11, 12, 13, 14), diverts triglycerides to exercising muscle during exercise and to brown adipose tissue during cold (15, 16), and prevents lipotoxicity in lymphatic macrophages (17). ANGPTL4 is also expressed in the intestine and a previous study from Mattijssen *et al.* found that ANGPTL4 knockout mice absorbed dietary fat at a faster rate than wild-type mice (18). They also found evidence that ANGPTL4 could inhibit pancreatic lipase in vitro and that a high-fat diet increased *Angptl4* expression (18). These data suggested that perhaps intestinal ANGPTL4 is induced by a high-fat diet, leading to inhibition of pancreatic lipase and decreased fat absorption. However, given the pleiotropic role of ANGPTL4 in lipoprotein metabolism and triglyceride clearance, determining the physiological importance of ANGPTL4 activity in the intestine has not been possible using a global ANGPTL4 knockout mouse.

In this study, we generated mice that lacked ANGPTL4 in intestinal epithelial cells to understand the role of intestinal ANGPTL4 in postprandial triglyceride absorption. We tested the hypothesis that in the absence of intestinal ANGPTL4, mice on high-fat diets would have a higher rate of postprandial fat absorption, become more obese, develop fatty liver, and become more insulin resistant.

## Materials and methods

### Mice

All animal procedures were approved by the Institutional Animal Care and Use Committee at the University of Iowa and were carried out in accordance with the National Institutes of Health Guide for the Care and Use of Laboratory Animals. Mice were group housed in a controlled environment with a 12/12 light–dark cycle, with food and water provided ad libitum during non-fasting conditions. During fasting conditions, water was provided ad libitum.

Generation of mice with floxed alleles of the *Angptl4* gene was described previously (14). Intestinal epithelial cell–specific knockout mice were generated by breeding *Angptl4*^fl/fl^ and Vil-cre mice (Jackson Labs, strain #021504). Both the floxed mice and the Vil-cre mice were on a C57Bl/6 background. At 12 weeks of age, *Angptl4*^flox/flox^ (fl/fl) and *Angptl4*^flox/flox^; Vil-Cre+ (IKO) littermate mice were assigned and fed a normal chow diet, a 45% high-fat diet (Research Diets D12451), or a 60% high-fat diet (Research Diets D12492). Eight mice of each sex were assigned to each cohort. Mice were fed their respective diets until the time of sacrifice (1 day on diet, 1 week on diet, or 12 weeks on diet). Mice who were on diet for 12 weeks were weighed weekly.

### RNA extraction and qPCR analysis

Mouse livers and small intestines were harvested and snap frozen in liquid nitrogen and stored at −80°C until processed. Tissues were pulverized before homogenization. For the small intestine samples, the entire length of the small intestine was homogenized for RNA extraction. Total RNA was extracted using Trizol (Invitrogen) according to manufacturer’s instructions. 2 μg of RNA was used to prepare cDNA with the High-Capacity cDNA Reverse Transcription kit (Applied Biosystems, 4368813) according to manufacturer’s instructions. Prepared cDNA was used for qPCR analysis using primers for *Angptl4* (forward-TTTGCAGACTCAGCTCAAGG; reverse-TCCATTGTCTAGGTGCGTGG) and *CycloA* (forward-TGGCAAGACCAGCAAGAA; reverse-CTCCTGAGCTACAGAAGGAATG). Prepared and diluted cDNA, primers, and SYBR Green ER qPCR Supermix reagent (Invitrogen, 11762100) were combined, and PCR was performed on the QuantStudio 6 Flex system (3 technical replicates per mouse, Applied Biosystems, Iowa Institute of Human Genetics). Gene expression was calculated using the ΔΔct method (19) using CycloA as the reference gene.

### Body composition analysis

Body composition [fat mass (g) and lean mass (g)] was measured by nuclear magnetic resonance (NMR) in mice at 18 weeks and 24 weeks of age (6 and 12 weeks on diet) after a 4 h fast. Mice were weighed immediately before being placed in a restraint tube and scanned with a Bruker LF50. NMR measurements were performed in the Fraternal Order of the Eagles Diabetes Research Center Metabolic Phenotyping Core at the University of Iowa.

### Glucose and insulin tolerance tests

Glucose tolerance tests (GTT) were performed at 22 weeks of age (10 weeks on diet). After a 6 h fast (beginning at the start of the light cycle), blood glucose was measured with a glucometer (OneTouch Ultra) before and 30, 60, 90, and 120 min after an intraperitoneal injection of glucose (1 g/kg). Blood samples were collected at 0, 30, 60, 90, and 120 min following injection. Blood glucose readings were measured at 0, 30, 60, 90, and 120 min following glucose injection using a glucometer (OneTouch Ultra). Insulin tolerance tests (ITT) were performed at 23 weeks of age (11 weeks on diet). After a 4 h fast, blood glucose was measured with a glucometer (OneTouch Ultra) before and 15, 30, 60, and 90 min after an intraperitoneal injection with insulin (0.5 U/kg, Humalin-R 100).

### Triglyceride measurements

Plasma was collected in fasted mice (6 h beginning at the start of the light cycle) at the ages indicated in the figures. Blood was collected into EDTA-coated capillary tubes and plasma was collected following centrifugation (1,500xg, 15 min, 4°C). Plasma triglycerides were analyzed in duplicate using the Infinity Triglyceride Reagent (Thermo Scientific) as previously described (14).

Liver triglyceride measurement was performed using a modified Folch extraction method (20). Liver tissues (approximately 100 mg) were homogenized in 500 μl of ice-cold PBS. 1.5 ml of a 2:1 (chloroform:methanol) mixture was added to each sample. Samples were vortexed briefly and incubated for 10–15 min. Samples were then centrifuged for 10 min at 2,050×g at 4°C. The lower organic phase was separated with a glass Pasteur pipette and placed into a 7 ml plastic vial (RPI 125509) and allowed to dry. The dried lipid was then dissolved with 500 μl of 2% Triton X-100 in chloroform and allowed to dry. The dried lipid was resuspended in 100 μl of molecular grade water. The sample was diluted 1 to 10 for triglyceride assay analysis. Analysis was performed using 2 μl of diluted lipid (technical duplicates) which was combined with Infinity Triglyceride Reagent (200 μl, Thermo Scientific TR22421) in a 96-well plate. The plate was incubated for 10 min at 37°C, gently tapped to ensure proper mixing of reagent with sample, and absorbance was read at 492 nm and 650 nm. Triglyceride concentrations were determined using a standard curve prepared from Triolein standard (Nu-Chek Prep, Lot T-235-N13-Y). Triglyceride levels were normalized to milligrams of tissue.

### Postprandial triglyceride absorption assays

After 1 day, 1 week, or 12 weeks on diet, mice were fasted for 4 h beginning at the start of the light cycle and injected retro-orbitally with 500 mg/kg body weight Tyloxapol to block triglyceride clearance. Mice were then gavaged with 20 μCi of [9,10-^3^H(N)]-Triolein (PerkinElmer, NET431001MC) in 100 μl of olive oil. Blood was collected 30, 60, 120, 180, and 240 min following injection and assayed for radioactivity in BioSafe II scintillation fluid (10 μl plasma/time point). After the 240-min time point, mice were sacrificed, and the small intestine was removed and perfused with PBS to remove the luminal contents. ∼1-inch pieces of the middle, proximal end, and distal end of the small intestine were removed and weighed. Chloroform:methanol (2:1) lipid extraction was performed on 40–90 mg of each tissue. After overnight incubation at 4°C and following phase separation with 1 ml of 2M CaCl_2_ the organic and aqueous phases were assayed for radioactivity using a Beckman-Coulter Scintillation Counter. The counts per million (CPMs) from each fraction were combined to give total uptake of CPMs. CPMs per gram of tissue was calculated for each section.

### Intestinal lipase activity assays

After an overnight fast (∼4 pm–8 am) and a 2 h refeeding, the luminal fluid of the small intestine was collected by dissecting out the small intestine and flushing it with 500–1000 μl of PBS. Luminal fluid and wash were collected and then centrifuged for 15 min at 5000 x g. The supernatant was collected and stored at −80°C. To assay for triglyceride lipase activity, protein levels for each sample were measured using the Biorad DC protein assay. Each sample was diluted to 5 μg/ml in PBS. 50 μl of diluted sample was mixed with 50 μl of substrate (0.84 μM EnzChek Lipase Substrate, 8 mM sodium taurocholate, 50 mM Tris-HCl pH 7.4, 140 mM NaCl, 2 nM CaCl_2_). Fluorescence (Excitation 485 nm; Emission 535 nm) was read every 2 min for 40 min at 37°C using an Infinite F200 plate reader (Tecan). Relative triglyceride lipase activity was calculated by determining the slope of the linear part of the curve (typically in the range between 10 to 25 min) and then subtracting the slope of the blank (sample with no luminal fluid). Technical duplicates were run for each sample. Points on graphs were plotted as a fold-change in activity compared to *Angptl4*^fl/fl^ mice fed a normal chow diet.

### Statistics

Statistics were performed using Graphpad Prism 10. Statistical tests used for each data set are listed in the respective figure legend.

## Results

### Experimental design

**Fig. 1:**
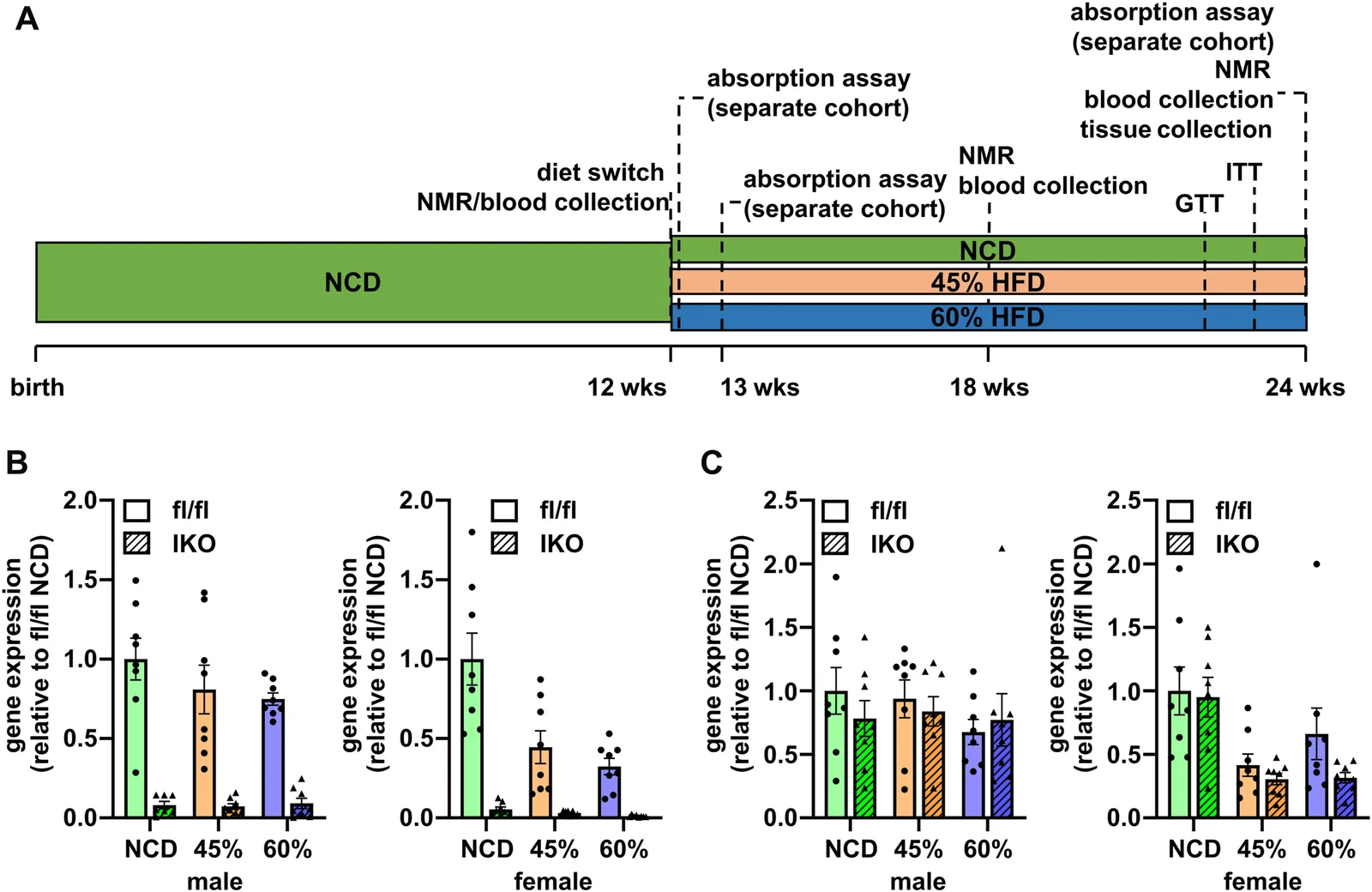
Experimental timeline and gene expression. A: Timeline of experimental procedures. Each group had 8 female and 8 male mice per diet. B-C: *Angptl4* gene expression in intestine (B) and liver (C) of male and female *Angptl4*^flox/flox^ (fl/fl) and *Angptl4*^flox/flox^; Vil-Cre+ (IKO) mice. Bars represent mean ± SEM. Circles and triangles represent individual mice.

The experimental design for this study is shown in Fig. 1A. We generated intestinal epithelial cell–specific ANGPTL4 knockout mice (IKO) by crossing *Angptl4* flox mice (14) with Villin-Cre mice. Throughout the study, flox/flox littermates (which lacked Cre) were used as controls. Male and female ANGPTL4 IKO and flox/flox littermates were fed a normal chow diet for 12 weeks and then randomly assigned one of three diets, a normal chow diet, a 45% (by kcal) high-fat diet, or a 60% high-fat diet. Mice were fed their respective diet for up to 12 weeks. We used 8 male and 8 female mice for each genotype. Gene expression data showed less than 10% residual intestinal *Angptl4* expression in IKO mice (Fig. 1B), indicating that most of intestinal ANGPTL4 is expressed in intestinal epithelial cells. Residual *Angptl4* expression likely comes from other cell types in the intestine, with macrophages being the most likely candidates as *Angptl4* can be expressed highly in macrophages (21). *Angptl4* expression in the liver was not significantly different than that of flox/flox littermates (Fig. 1C). Interestingly, we did not observe an increase in intestinal *Angptl4* expression after 12 weeks of high-fat feeding (Fig. 1B).

### Bodyweight and body composition in ANGPTL4 IKO mice

**Fig. 2:**
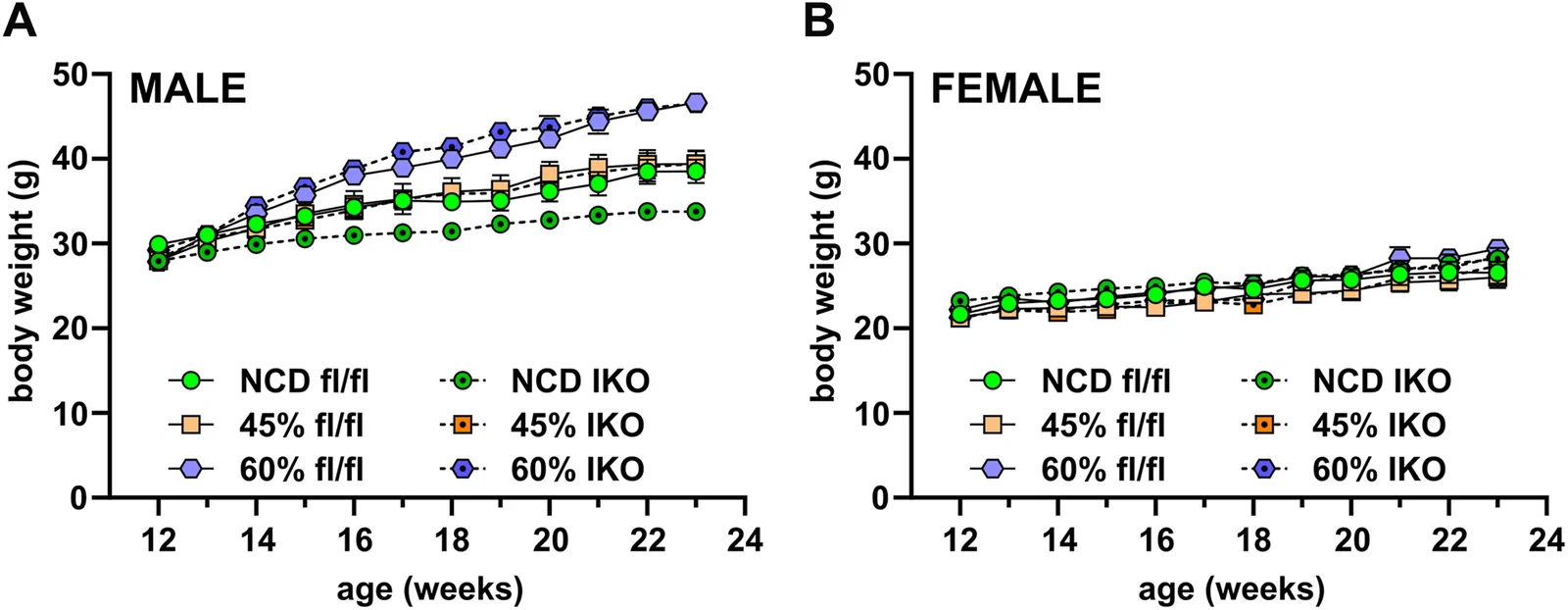
Body weight for male and female mice. A-B: Body weights of male (A) and female (B) *Angptl4*^flox/flox^ (fl/fl) and *Angptl4*^flox/flox^; Vil-Cre+ (IKO) mice fed a normal chow (NCD), 45% high-fat (45%), or 60% high-fat (60%) for 12 weeks.

After initiation of high-fat feeding, body weight was measured weekly. We hypothesized that the lack of intestinal epithelial cell ANGPTL4 would lead to increased fat absorption and possibly increased weight gain. This, however, was not the case. Body weights for ANGPTL4 IKO mice were similar to flox/flox littermates across sex and diet except for male mice on a normal chow diet, where the male ANGPTL4 IKO mice had slightly lower body weights than flox/flox mice (Fig. 2).

**Fig. 3:**
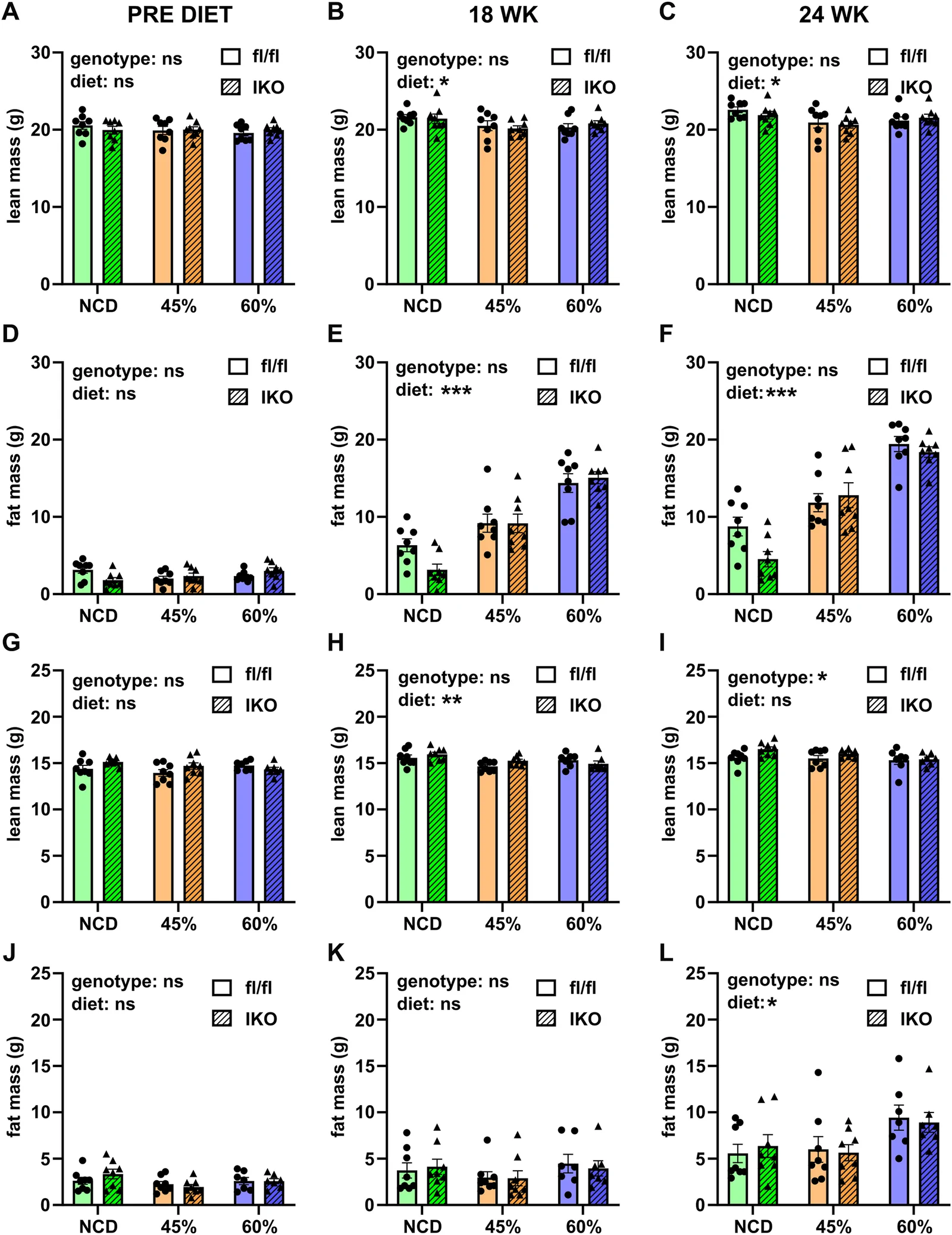
Body composition across diet and genotype. Lean (A-C, G-I) and fat (D-F, J-L) mass in male (A–F) and female (G–L) *Angptl4*^flox/flox^ (fl/fl) and *Angptl4*^flox/flox^; Vil-Cre+ (IKO) mice as measured by NMR. Bars represent mean ± SEM. Circles and triangles represent individual mice. ns = not significant, ∗*P* < 0.05, ∗∗*P* < 0.01, ∗∗∗*P* < 0.001 by 2-way ANOVA.

Similarly, there was little difference in body composition between flox/flox and ANGPTL4 IKO mice (Fig. 3). As expected, fat mass in males was higher in diets with higher fat content (Fig. 3D–F). Female mice showed little differences in body weights on the different diets (Fig. 2B) and only slight difference in fat mass at 24 weeks of age (Fig. 3L).

### Glucose and insulin tolerance

**Fig. 4:**
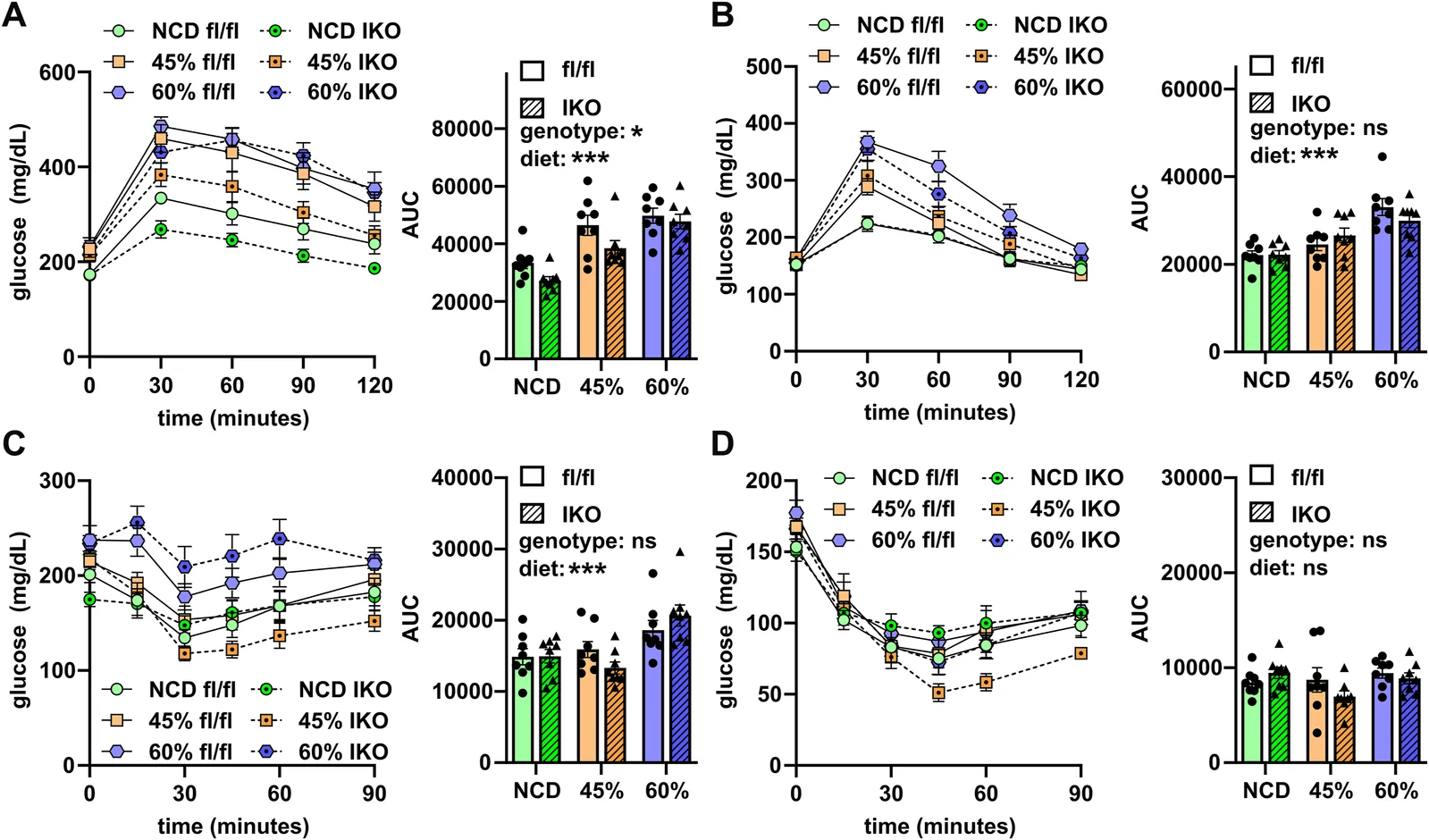
Glucose and insulin tolerance tests. A-B: Glucose tolerance tests were performed on fasted (6 h) male (A) and female (B) *Angptl4*^flox/flox^ (fl/fl) and *Angptl4*^flox/flox^; Vil-Cre+ (IKO) mice at 22 weeks of age. Mice were injected with glucose (1 g/kg) and blood glucose concentrations were measured over 2 h. Points represent glucose levels (means ± SEM) at each respective time point. Bar graphs show area under the curve (AUC). C-D: Insulin tolerance tests were performed in fasted (4 h) male (C) and female (D) *Angptl4*^flox/flox^ (fl/fl) and *Angptl4*^flox/flox^; Vil-Cre+ (IKO) mice at 23 weeks of age. Mice were injected with 0.5 U/ml of human insulin (Humalin-R) and blood glucose concentrations were measured over 90 min. Points represent glucose levels (means ± SEM) at each respective time point. Bar graphs show area under the curve (AUC). Circles and triangles represent individual mice. ns = not significant, ∗*P* < 0.05, ∗∗∗*P* < 0.001 by 2-way ANOVA.

High-fat diet feeding decreased glucose tolerance in both male and female mice (Fig. 4A, B). Contrary to our initial hypothesis, glucose tolerance was slightly improved in male ANGPTL4 IKO mice (Fig. 4A), but there were no genotype-specific differences in female mice (Fig. 4B). There were no significant genotype-specific differences in insulin tolerance for either male or female mice (Fig. 4C, D).

### Triglyceride levels

**Fig. 5:**
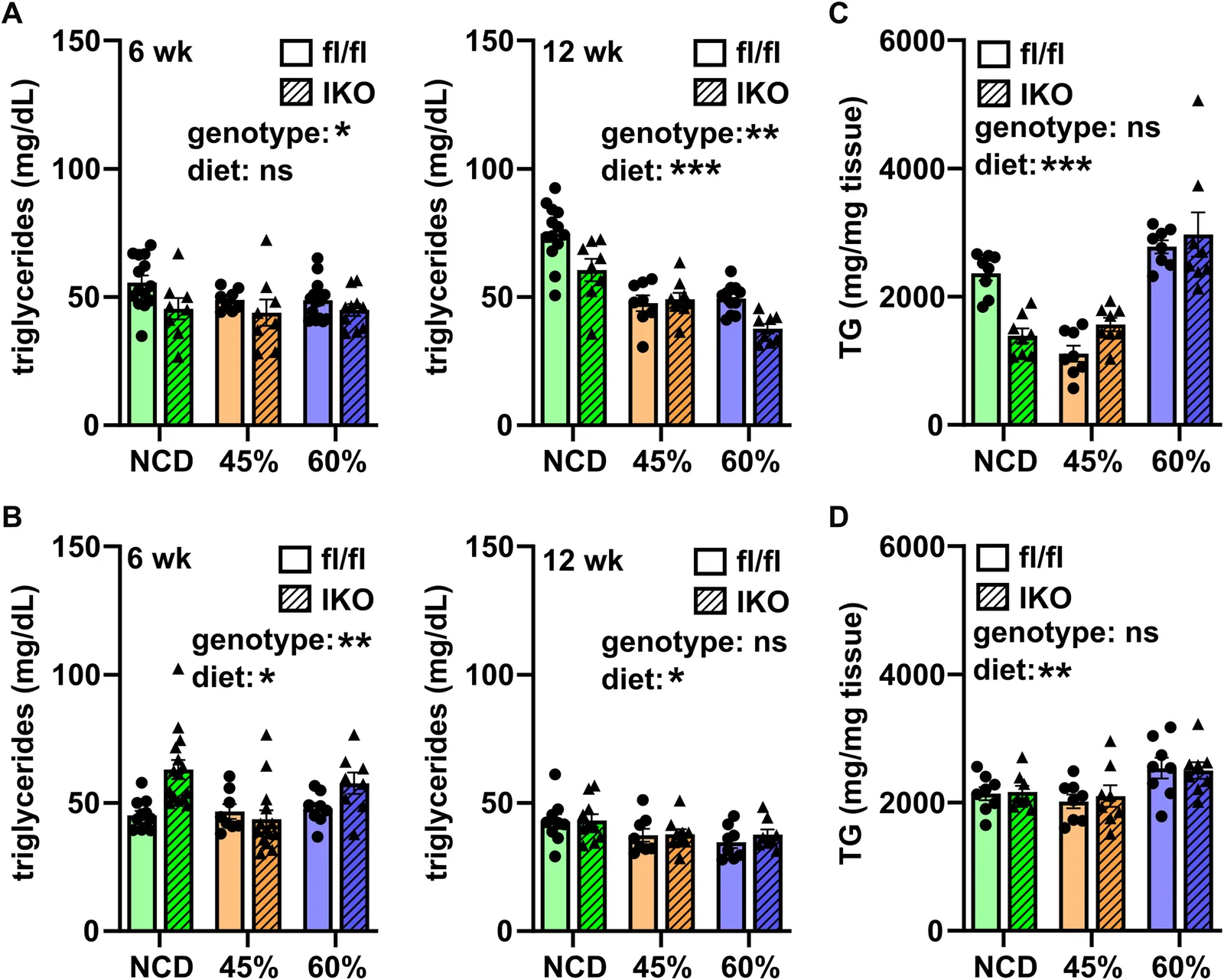
Fasting plasma and liver triglyceride levels. A-B: Plasma was collected from mice following a 6 h fast. Data shows plasma triglyceride levels as measured in male (A) and female (B) *Angptl4*^flox/flox^ (fl/fl) and *Angptl4*^flox/flox^; Vil-Cre+ (IKO) mice at the indicated time on diet. C-D: Liver triglyceride measurement of male (C) and female (D) *Angptl4*^flox/flox^ (fl/fl) and *Angptl4*^flox/flox^; Vil-Cre+ (IKO) mice at 24 weeks of age (12 weeks on diet). Bars represent mean ± SEM. Circles and triangles represent individual mice. ns = not significant, ∗*P* < 0.05, ∗∗*P* < 0.01, ∗∗∗*P* < 0.001 by 2-way ANOVA.

We hypothesized that intestinal epithelial cell ANGPTL4 deficiency would lead to higher plasma and liver triglyceride levels due to higher fat absorption. This was not the case. In male mice, plasma triglyceride levels tended to be lower in ANGPTL4 IKO mice (Fig. 5A), while in females plasma TGs were slightly higher after 6 weeks on diet for NCD and 60% HFD-fed mice, and not different at all after 12 weeks on any diet (Fig. 5B). There were no genotype-specific differences in liver triglycerides in either sex, though unsurprisingly mice fed a 60% high-fat diet had higher liver TGs than mice fed the other diets (Fig. 5C, D).

### Postprandial triglyceride absorption and intestinal lipase activity

Our initial hypothesis was that intestinal epithelial cell ANGPTL4 deficiency would increase activity of pancreatic lipase and increase fat absorption, thus increasing body weight and plasma and liver triglycerides. As body weights and triglyceride levels did not increase as expected, we next tested postprandial triglyceride absorption, entry of dietary fat into the circulation, and intestinal lipase activity. Flox/flox and IKO mice were fed a normal chow diet, a 45% HFD, or a 60% HFD for 1 day, 1 week or 12 weeks. After injection with tyloxapol to prevent clearance of radiolabel from the circulation, the rate of postprandial triglyceride absorption was assayed by gavaging mice with radiolabeled triolein and measuring appearance of radiolabel in the blood.

**Fig. 6:**
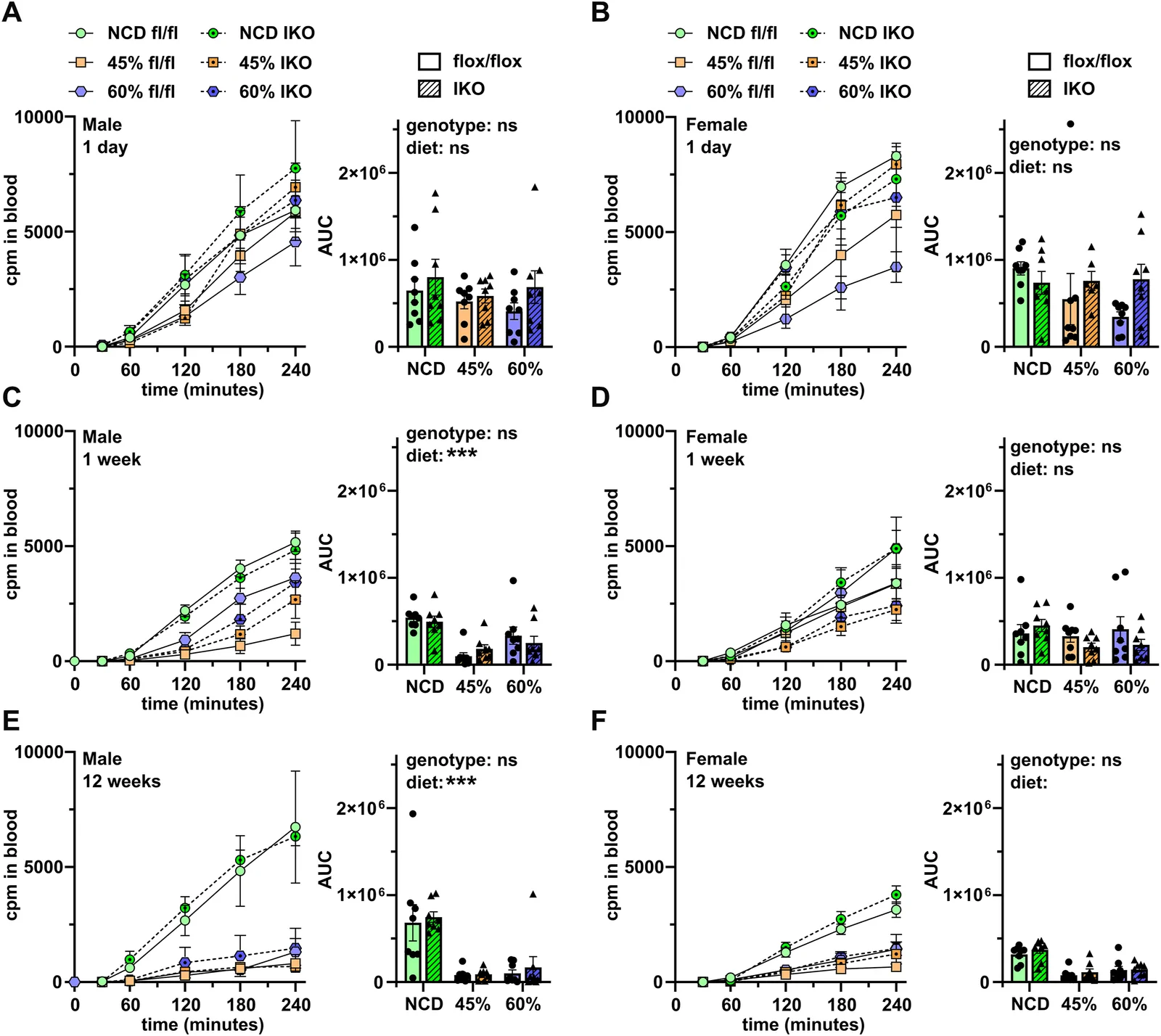
Postprandial triglyceride absorption tests. Male (A, C, E) and female (B, D, F) *Angptl4*^flox/flox^ (fl/fl) and *Angptl4*^flox/flox^; Vil-Cre+ (IKO) mice fed a normal chow (NCD), 45% high-fat (45%), or 60% high-fat (60%) for 1 day (A, B), 1 week (C, D), or 12 weeks (E, F). Mice were then fasted 4 h and injected with Tyloxapol (500 mg/kg) to block triglyceride clearance. Mice were then gavaged with ^3^H-triolein in olive oil. Blood was collected 30, 60, 120, 180, and 240 min after gavage and assayed for ^3^H levels. Points represent CPMs (means ± SEM) at each respective time point. Bar graphs show area under the curve (AUC). Circles and triangles represent individual mice. ns = not significant, ∗∗∗*P* < 0.001 by 2-way ANOVA.

After one day of high-fat feeding, there were no significant differences in the rate of postprandial triglyceride absorption across diets or genotypes in either male or female mice (Fig. 6A, B). After a week on diet, the rate of postprandial triglyceride absorption in male mice fed a high-fat diet was lower than that of those fed a normal chow diet (Fig. 6C, D). After 12 weeks on diet, the rate of postprandial triglyceride absorption was strikingly lower in both male and female mice fed a high fat diet compared to those fed a normal chow diet (Fig. 6E, F). At no time point were IKO mice significantly different from flox/flox mice (Fig. 6).

**Fig. 7:**
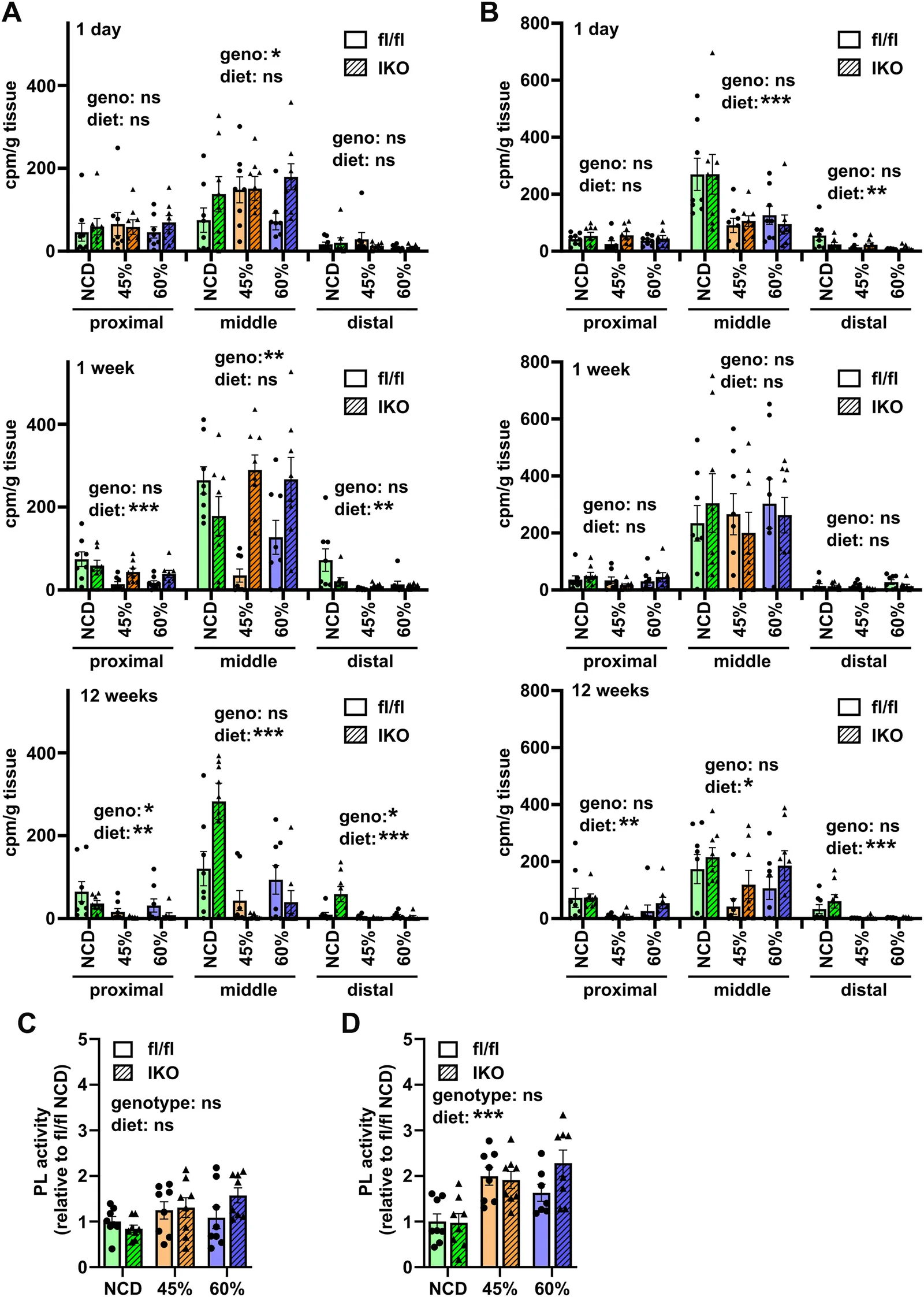
Intestinal triglyceride uptake and luminal lipase activity. Male (A) and female (B) *Angptl4*^flox/flox^ (fl/fl) and *Angptl4*^flox/flox^; Vil-Cre+ (IKO) mice were fed a normal chow (NCD), 45% high-fat (45%), or 60% high-fat (60%) for 1 day, 1 week, or 12 weeks. Mice were then fasted 4 h and injected with Tyloxapol (500 mg/kg) to block triglyceride clearance and then gavaged with ^3^H-triolein in olive oil. After 4 h, proximal, middle, and distal sections of the small intestine were collected, washed, and assayed for ^3^H levels. C-D: At 24 weeks of age (12 weeks on diet), the intestinal fluid of male (C) and female (D) *Angptl4*^flox/flox^ (fl/fl) and *Angptl4*^flox/flox^; Vil-Cre+ (IKO) mice fed a normal chow (NCD), 45% high-fat (45%), or 60% high-fat (60%) was collected and assayed for triglyceride lipase activity. Bars represent mean ± SEM. Circles and triangles represent individual mice. ns = not significant, ∗*P* < 0.05, ∗∗*P* < 0.01, ∗∗∗*P* < 0.001 by 2-way ANOVA.

To determine if differences in the rate of postprandial triglyceride absorption were due to differences in intestinal uptake or intestinal secretion of triglyceride, we also collected intestinal tissue from proximal, middle, and distal sections of the small intestine and measured radiolabel levels. Overall, radiolabel levels were highly variable, but we did not observe any consistent differences between genotypes. We did find that after 12 weeks on diet, mice fed either of the high fat diets had significantly less radiolabel in the intestinal tissue than NCD-fed mice (Fig. 7A, B). These data suggest lower intestinal uptake of dietary fat in the mice fed high-fat diets. The lower rate of postprandial triglyceride absorption on the high-fat diets did not seem to be a result of lowered pancreatic lipase activity as lipase activity from the lumen of the intestine was not lower in mice fed a high-fat diet, and in females it was significantly higher (Fig. 7C, D).

## Discussion

In this study we tested the hypothesis that, in the absence of intestinal ANGPTL4, mice on high-fat diets would have greater pancreatic lipase activity, a higher rate of postprandial fat absorption, become more obese, develop fatty liver, and become more insulin resistant. Our data did not support this hypothesis, and we found no indication that intestinal ANGPTL4 plays a major role in postprandial triglyceride absorption. We did not observe an increase in *Angptl4* gene expression with high-fat feeding, nor did intestinal epithelial cell–specific deletion of ANGPTL4 alter pancreatic lipase luminal activity or the rate of postprandial triglyceride absorption on any diet or with either sex. We did observe that chronic high-fat diet feeding decreased the rate of postprandial triglyceride absorption, but this decrease was completely independent of intestinal epithelial cell ANGPTL4.

Our initial goal in this study was to extend the findings of Mattijssen *et al.* (18), which suggested a role for intestinal ANGPTL4 in dietary fat absorption. Instead, we found little evidence that intestinal ANGPTL4 regulates intestinal uptake of dietary fat. While our findings appear to contradict those of the earlier study, this may not necessarily be the case. Both our study with intestinal epithelial cell ANGPTL4 deficiency and the earlier study with whole-body ANGPTL4 deficiency found that ANGPTL4 deficiency did not alter the amount of radiolabel entering the circulation after a gavage of radiolabeled triglyceride (18). Where the studies differed was in the amount of radiolabel observed in the intestine and the levels of lipase activity in the intestinal lumen. Mattijssen *et al.* found that fat absorption into the intestine and luminal lipase activity were both increased with whole-body ANGPTL4 deficiency (18). Their conclusion, and the most parsimonious explanation of their data, was that ANGPTL4 inhibits pancreatic lipase and thus limits fat absorption. The fact that they did not observe increased levels of radiolabel in the blood was interesting but would not undermine their conclusion as it has been shown that not all dietary fat absorbed in the intestines gets immediately secreted as chylomicrons (22, 23, 24). However, their data does not preclude the possibility that their observations were the result of ANGPTL4 acting elsewhere, like in liver or in adipose tissue, to influence dietary fat absorption. It is tempting to speculate that the low circulating triglyceride levels that occur with ANGPTL4 deficiency (5, 11, 25, 26, 27, 28, 29) could drive increased intestinal fat absorption. However, little has been done to test this idea. One mouse model of low circulating triglyceride levels, the ApoC3-deficient mouse, has been tested for intestinal fat absorption. Jong *et al.* reported that while ApoC3-deficient mice have less than a third of the fasting plasma triglyceride levels of wild-type mice, lipid absorption was normal compared to control mice (30). However, when ApoC3-deficient mice were crossed with ApoE-deficient mice, dietary fat absorption was increased compared to ApoE-deficient mice with normal ApoC3 (30). Importantly, in their study, the rate of postprandial triglyceride absorption was measured only by measuring radiolabel entry into the circulation. Radiolabel uptake into the intestine was not measured, leaving open the possibility that low plasma triglyceride levels somehow altered dietary triglyceride uptake into the intestine. While we found no change in the rate of postprandial triglyceride absorption when ANGPTL4 was absent from intestinal epithelial cells, we cannot definitively exclude a role for ANGPTL4 in fat absorption or in the regulation of pancreatic lipase. It is possible that ANGPTL4 normally acts to regulate intestinal lipase activity but that in the absence of ANGPTL4 compensatory mechanisms exactly preserve the rate of postprandial triglyceride absorption. What this compensatory mechanism would be is not clear. Neither ANGPTL3 nor ANGPTL8, the other ANGPTL proteins known to inhibit lipases, are expressed in the intestine (9, 25, 31, 32).

If ANGPTL4 does not inhibit intestinal lipases, what then is the role of ANGPTL4 in the intestine? One intriguing possibility is that rather than regulating the uptake of triglycerides from the intestinal lumen, ANGPTL4 regulates the uptake of triglycerides from the circulation into enterocytes. It has previously been shown in animal models that the intestine takes up fatty acids, including triglyceride-derived fatty acids from the circulation (33, 34, 35, 36). Moreover, fatty acids taken up from the circulation appear to be a separate pool and have separate functions from those absorbed from the intestinal lumen, functions that may include the regulation of chylomicron secretion (37, 33, 34, 35, 36). Conceivably, ANGPTL4 could alter the uptake of fatty acids into enterocytes through its well-established ability to inhibit LPL, the enzyme responsible for hydrolyzing lipoprotein triglycerides. However, there is little expression of LPL in the intestine (38, 39). While there are cases where LPL protein localizes to tissues where it is not expressed (40), such a phenomenon has not been described for the intestine. Alternatively, ANGPTL4 secreted from intestinal epithelial cells into the vasculature could regulate LPL in other tissues. One of the early studies examining the effects of the gut microbiota on obesity found that *Angptl4* expression was suppressed specifically in the intestine by the microbiota of conventionalized mice and that this suppression correlated with an increase in LPL activity in adipose tissue (41). It is not clear whether ANGPTL4 secreted from the intestine would have a significant effect on LPL activity in other tissues given that the ANGPTL4 secreted from the liver, which has much higher expression of *Angptl4* than the intestine, appears to have little effect on systemic LPL activity (42). We did see a small decrease in plasma triglycerides in male mice that lacked intestinal epithelial cell ANGPTL4, which could indicate some effect of intestinal ANGPTL4 on plasma TG clearance, but we did not see a decrease in circulating triglycerides in female mice. It is important to note that as the rate of triglyceride absorption was measured after blocking triglyceride clearance with tyloxapol, we would not have been able to detect differences in basolateral triglyceride uptake in the intestinal epithelial cell–specific ANGPTL4 deficient mice, nor would we observe changes in LPL-mediated uptake into any other tissues. Future experiments could test for differences in systemic triglyceride uptake or LPL activity.

Of course, the primary role of ANGPTL4 in the intestine may be independent of lipase inhibition. ANGPTL4 has been reported to play roles in intestinal permeability (43, 44) and inflammation (43, 44, 45, 46, 47). In fact, a very recent study from Chua *et al.* used intestinal epithelial cell–specific ANGPTL4 deficient mice to show that loss of ANGPTL4 in the intestine protects against diet-induced gut permeability and vascular leakiness, preserving expression of tight junction and endothelial markers of barrier function (44). Their data suggest that the major role of ANGPTL4 in the intestine may be the modulation of intestinal permeability rather than lipase inhibition.

Although we did not observe a role for intestinal epithelial cell ANGPTL4 in postprandial triglyceride absorption on any of the diets we tested, we did find that chronic high fat feeding reduced the rate of postprandial triglyceride absorption. This was especially evident after 12 weeks on diet, when both male and female mice fed either a 45% or 60% high-fat diet displayed strikingly less entry of radiolabeled fatty acid into the bloodstream compared to mice fed normal chow. The reduced appearance in the blood stream could not be explained by retention of triglycerides in the enterocytes as we did not observe increased levels of radiolabel in the intestine. Decreased rates of postprandial triglyceride absorption after a high-fat diet have been reported previously (48, 49). In these studies, the rate of triglyceride appearance in the circulation after an oral gavage of olive oil was measured in mice fed a high fat diet for 3 (48) or 9 (49) weeks. Similar to our findings here, these studies found that the rate of postprandial triglyceride absorption was strikingly lower in mice fed a high-fat diet (48, 49). Interestingly, Douglass *et al.* found that the reduction in postprandial absorption rate occurred prior to changes in body weight (48), consistent with our findings here. Moreover, the study from Uchida *et al.* found that despite the reduced rate of triglyceride appearance in the circulation, the enterocytes of high-fat–fed mice did not appear to retain more triglycerides than those of chow-fed mice (49), also consistent with what we report here. The mechanism by which chronic high-fat diet feeding reduces the rate of postprandial triglyceride absorption is unclear. The most straightforward mechanism would be a reduction in intestinal triglyceride lipase activity. However, when we measured luminal lipase activity, we did not observe a decrease. It remains possible that while lipase activity is not decreased, lipolysis is decreased in some way, for example, by a decrease in bile acid secretion. It is also possible that dietary fats are broken down normally, but the uptake of fatty acids into enterocytes is reduced. One major limitation of our study is that we did not do a thorough analysis of fecal lipid content. Such an analysis could shed light on the fate of dietary triglycerides in HFD-fed mice. We did attempt to measure fecal radiolabel during our fat absorption assays. However, the levels of radiolabel were so variable and inconsistent that we have not reported the data here.

Our study has additional limitations. Although we found that intestinal epithelial cell–specific ANGPTL4 deficiency had little effect on the rate of postprandial triglyceride absorption or luminal lipase activity, we did not directly test the ability of ANGPTL4 to inhibit pancreatic lipase. Previous studies have shown that ANGPTL4 can inhibit pancreatic lipase, although the concentrations needed to inhibit pancreatic lipase appear to be much higher than those needed to inhibit LPL (18, 50). Another limitation is that we only examined postprandial triglyceride absorption in mice. While mice and humans share the same basic mechanisms of fat absorption, including the crucial role of pancreatic lipase, and while fat absorption is known to decrease with age in both mice and humans (51, 52, 53), it is certainly possible that the HFD-induced reduction in the rate of postprandial triglyceride absorption we observed with mice is a phenomenon not shared with humans.

In summary, we found that intestinal epithelial cell–specific ANGPTL4 deficiency does not seem to alter rate of postprandial triglyceride absorption. In contrast, the rate of postprandial triglyceride absorption is reduced in mice fed a chronic high-fat diet.

## Data availability

The data that support the findings of this study are available from the corresponding author upon reasonable request.

## Conflict of interest

The authors declare that they have no conflicts of interest with the contents of this article.
